# Intense Laser Pulse Interaction With Graphene and Graphene Ribbons

**DOI:** 10.3389/fchem.2022.859405

**Published:** 2022-04-25

**Authors:** F. H. M. Faisal

**Affiliations:** Department of Physics, Universität Bielefeld, Bielefeld, Germany

**Keywords:** graphene, ribbon, adiabatic-Hamiltonian, energy-bands, population, current, CEP, laser-pulse

## Abstract

In this work we investigate quantum mechanically the interaction of an intense ultrashort laser pulse with the graphene monolayer as well as with the armchair graphene ribbons of different widths. We consider a tight binding (TB) Hamiltonian of the monolayer graphene and give two rules for deriving the dispersion relations of the armchair graphene *ribbons* of any width, N, from the TB eigenvalues of the *monolayer*. The band structure of the monolayer and the armchair ribbons of different widths are discussed with illustrations. The time-dependent wavefunctions of the systems and the expectation values of interest are determined by solving the coupled equations of the band amplitudes “exactly” (numerically). First, simulations are made for the *population* excitation in the conduction band (CB) from the valence band (BV), the VB-CB *inter*band *correlation* (or “coherence”), the *intra*band, the *inter*band and the total *currents* in the monolayer graphene. The graphene currents are compared with the corresponding currents induced in an armchair ribbon (width, N = 3). The change from the 2D monolayer to the 1D ribbon shows a remarkable transition of the dominance of the *intra*band current that leads to a near *steady* total current in the monolayer, to a dominance of the *inter*band current in the ribbon that induces an *oscillatory* current in the ribbon beyond the pulse duration. The difference observed might be a combined effect of the “confinement” in one dimention and a finite band-gap minimum in the case of the ribbon. However, this transition should be further investigated for better clarity. A brief comparison of the radiation spectra emitted from the monolayer and from the ribbon is also made. They show a grossly similar structure and a relative insensitivity with respect to the detailed structure of the targets chosen. This might be due to the dominance of virtual continuum-continuum transitions, to and from the bands states, that lie behind the fundamental quantum process of high harmonic emissions. Lastly, the dependence of the charge currents, induced in a *ribbon* of unit width (N = 1), on the carrier-envelope-phase (CEP) of the incident laser pulse is investigated. It is seen that the shape of the *main* part of the current produced in the ribbon can be fully reversed by changing the CEP of the ultrashort laser pulse from 0 to *π*. More generally, it is suggested that the pulse shape of the charge carriers in the ribbon could be designed by similarly tailoring the form of the *vector potential* of the incident laser pulse.

## 1 Introduction

Graphene is a two dimensional hexagonal one-atom-thin monolayer of carbon atoms that shows remarkable material, electrical and optical properties (e.g. Castro Neto et al. (2009)) with much potentials for future applications. The study of the interaction of intense laser light with monolayer graphene and theoretical and experimental investigations of the emission of high harmonic radiation from them began quite sometime ago (e.g., Faisal and Kaminski (1997); Ghimire et al. (2011); Faisal (2011); Faisal (2013); Schubert et al. (2014); Luu et al. (2015); Vampa et al. (2015); Liu et al. (2017); Sivis et al. (2017); Yoshikawa et al. (2017)). Investigations of the nature of electric currents in graphene generated by intense laser pulses have also made considerable progress (e.g. Kelardeh et al. (2015), Higuchi et al. (2017), Ernotte, et al. (2018), Heide et al. (2020). For example, ballistic electric currents and sub-optical cycle “Stueckelberg oscillations” in graphene monolayers induced by ultrashort lasers have been observed recently by Hommelhoff and collaborators (Higuchi et al. (2017); Heide et al. (2020)). A ramarkable progress had been reported recently by Karakachian et al. (2020) in synthesising high quality armchair graphene *ribbons* having finite widths. Their method makes use of the side walls of 6H-SiC mesa structures to epitaxially grow the armchair ribbons of different widths on them, while the ARPES technique was used to investigate the electronic structure of the sub-bands of armchair ribbons that revealed the presence of *band gaps* in most ribbons as well as a gapless pair of bands for ribbons of certain widths (see, below). This development opens up new prospects of further research on the effect of quantum *confinement* and of potential applications of the ribbons in semiconductor electronics, specially, where the monolayer graphene is not directly usable due to its missing band gap.

In this work we explore (quantum mechanically) the interactions of an intense ultrashort laser pulse with the monolayer graphene *and* with armchair graphene ribbons. To this end first we consider an analytical TB (tight binding) Hamiltonian in the reciprocal lattice space, determine its eigenvalues and eigenfunctions, give two simple rules for constructing the band-system of armchair ribbons (of any width) from the graphene bands and, briefly illustrate the lattice structure of the monolayer graphene and of the armchair ribbons. Next, the current and the transition dipole operators (relevant for the study of laser interactions) are constructed analytically from the TB Hamiltonian. The laser interaction is introduced using the *minimal coupling* prescription in the reciprocal lattice space and the time-dependent Schrödinger equation of the interacting system is obtained in the adiabatic representation (cf. Faisal (2011)). To solve the equation, we expand the total wavefunction in terms of the adiabatic eigenstates of the “instantaneous” Hamiltonian and construct a pair of coupled dynamical equations (cf. Faisal (2016)) for the occupation amplitudes of the valence band (VB) and the conduction band (CB) of the interacting system. The equations are integrated numerically “exactly” to simulate the transition probabilities and the expectation values of the observables of the present interest. They are used to investigate 1) the transfer of population from the VB to the CB, 2) the induced VB-CB correlation (or “coherence”), 3) the ultrashort charge-currents generated both in the monolayer graphene and in the armchair graphene ribbons, as well as, 4) the radiation emitted from the generated charge-currents. Finally, the possibility of controlling the shape of the generated ultrashort charge-currents by choosing the incident laser pulse suitably is also considered for the case of an armchair ribbon of unit width (N = 1). The results of the simulations for the monolayer and the ribbons are presented, compared and discussed, with graphical illustrations.

## 2 Theoretical Model

**FIGURE 1:**
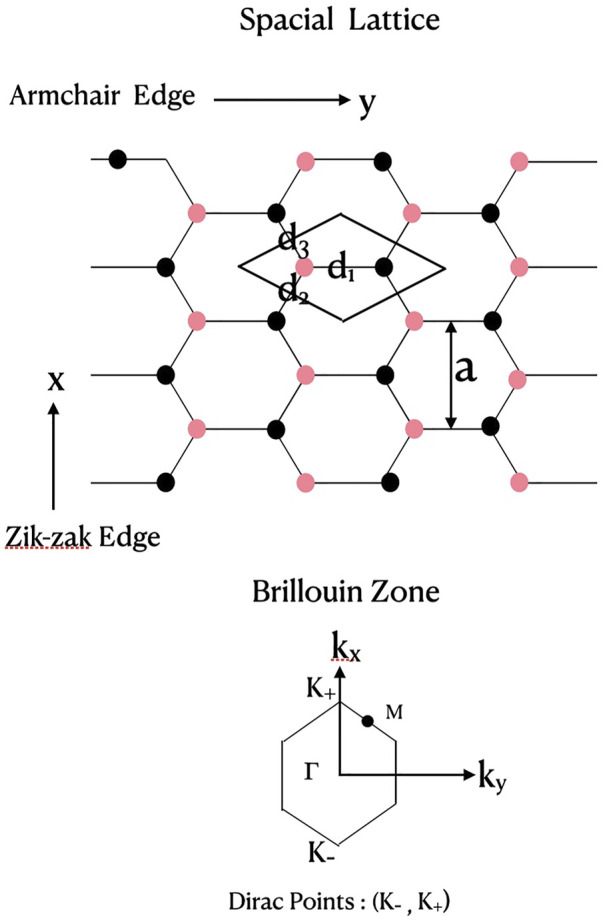
Lattice structure of a graphene monolayer. The upper part shows a 2D hexagonal carbon lattice with two (red, black) interpenetrating triangular Bravais sub-lattices. The unit cell (rhombus) has two atoms per cell (red, black). The nearest neighbour distances from one atom (red) are (**d**_1_, **d**_2_, **d**_3_); *a*_*CC*_ is carbon-carbon bond length, $a=\sqrt{3}a_{CC}$ = lattice constant = 0.246 nm = 4.649 [a.u.]. Note the “armchair” structure along the horizontal axis and the “zik-zak” structure along the vertical axis. The lower part of the figure shows the first Brillouin zone with symmetry points (Γ, *M*) and the Dirac points (*K*_−_, *K*_+_).

The lattice structure of a two dimensional graphene monolayer is illustrated schematically in Figure 1. The upper part of the figure shows the hexagonal honeycomb carbon lattice with two (red and black) interpenetrating triangular Bravais sub-lattices. A unit cell with two atoms per cell is also outlined (the rhombus in the figure). The 2D lattice vectors are defined by $a_{1}=\left\{a/2,\sqrt{3}a/2\right\},a_{2}=\left\{a/2,-\sqrt{3}a/2\right\}$ where, *a* = |**a**_1_| = |**a**_2_| is the lattice constant. The nearest neighbour (nn) distances from one atom (red) of the cell are $d_{1}=\left\{0,a/\sqrt{3}\right\},d_{2}=\left\{a/2,-a/\left(2\sqrt{3}\right)\right\},d_{3}=\left\{-a/2,-a/\left(2\sqrt{3}\right)\right\}$ . They define the complex “geometric” factor $h\left(k\right)=\sum_{j=1}^{3}e^{ik\cdot d_{j}}$ with, |**d**_1_| = |**d**_2_| = |**d**_2_| = *a*_*CC*_, the carbon-carbon bond length, and, **K**_±_ = ±{4*π*/(3*a*), 0} are the two non-equivalent “Dirac points” of band degeneracy (see, below). Note that the lattice constant $a=\sqrt{3}a_{CC}$ . The lower part of the figure shows the first Brillouin zone, along with the symmetry points Γ and *M*, as well as the Dirac points (*K*_−_, *K*_+_). The “armchair” structure of the horizontal edge and the zik-zak structure of the vertical edge of the monolayer graphene sheet are to be noted here.

**FIGURE 2:**
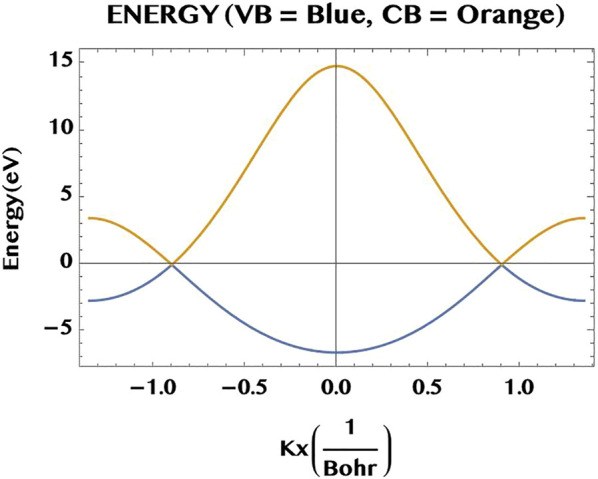
A 1D cut of the band structure of graphene monolayer along the *k*_*x*_-axis, with *k*_*y*_ =0. The lower lying valence band (VB, blue) and the upper lying conduction band (CB, orange) are degenerate at the two Dirac points (along the *k*_*x*_-axis at $\pm \frac{4\pi }{3a}=\pm 0.9\left[\frac{1}{Bohr}\right]$ , 1*Bohr* =0.0529 nm). Note that for a non-vanishing overlap integral *s*_0_ = 0.129, the bands are linear and symmetric near the Dirac point (but differ away from it).

The graphene monolayer is often theoretically modelled by a symmetric TB Hamiltonian near the Dirac points where the energy dispersion relations and the energy bands are linear and symmetric (see, e.g. review Castro Neto et al. (2009)). Most responses of graphene to weak static and/or low frequency fields are dominated by this domain of the Brillouin Zone (BZ). We point out that also for the laser fields in the near infrared wavelength (800 nm) and at an intensity of 1 TW/cm^2^, as used in this work, we tested (following the suggestion of an anonymous referee) and found *no* significant effect of the band asymmetry with non-zero overlap integral *s*_0_ = 0.129, as in this work, and *s*_0_ = 0 for the symmetric bands. This is apparently due to the high values of the dipole operator in the vicinity of the Dirac points (cf. Figure 6) where the bands are essentially symmetric and linear (cf. Figure 2).

Assuming two carbon atoms per unit cell (red and black dots in the rhombus of Figure 1), each described by a *p*_*z*_-orbital oriented perpendicular to the graphene plane and, taking only the nearest neighbour (nn) interactions, the stationary wavefunction *ψ*(**k**) of the graphene monolayer can be written as a linear combination of the two Bloch functions associated with the two *p*_*z*_ orbitals (per cell). We may then apply the variational principle as usual to construct the expectation value of the energy operator *H* ^0^, and arrive at the following 2 × 2 variational matrix equation (e.g. Saito et al. (1998); McCann (2012)) with respect to the two amplitudes associated with the two Bloch functions, *ψ*(**k**) = {*ψ*_1_(**k**), *ψ*_2_(**k**)} as well as the energy of the least bound *p*_*z*_ orbital, *ϵ*_2*p*_:

$$H^{0}\left(k\right)\psi \left(k\right)=ES\left(k\right)\psi \left(k\right)$$ (1)

where *H* ^0^(**k**) is a 2 × 2 matrix with elements

$$\begin{array}{ccc} H_{11}^{0}\left(k\right) & = & \epsilon_{2p}\\ H_{12}^{0}\left(k\right) & = & -g_{0}h\left(k\right)\\ H_{21}^{0}\left(k\right) & = & -g_{0}h^{*}\left(k\right)\\ H_{22}^{0}\left(k\right) & = & \epsilon_{2p} \end{array}$$ (2)

The complex geometric factor *h*(**k**) can be expressed by its real amplitude *f* and phase *χ* as:

$$\begin{array}{ccc} h\left(k\right) & = & f\left(k\right)e^{i\chi \left(k\right)}\\ f\left(k\right) & = & {\left(h_{1}^{2}\left(k\right)+h_{2}^{2}\left(k\right)\right)}^{1/2}\\ h_{1}\left(k\right) & = & 2\cos\left(\frac{k_{y}a}{2\sqrt{3}}\right)\cos\left(k_{x}a/2\right)\\ h_{2}\left(k\right) & = & \sin\left(k_{y}a/\sqrt{3}\right)-2\sin\left(\frac{k_{y}a}{2\sqrt{3}}\right)\cos\left(k_{x}a/2\right)\\ \chi \left(k\right) & = & \arctan\left(\frac{h_{2}\left(k\right)}{h_{1}\left(k\right)}\right) \end{array}$$ (3)

where, *g*_0_ is the “hopping” integral (the nearest neighbour interaction matrix element); the lattice constant $a=\sqrt{3}a_{CC}$ and, *a*_*CC*_ is the carbon-carbon bond length. The 2 × 2 overlap matrix *S* is defined by its elements

$$\begin{array}{ccc} S_{11} & = & 1\\ S_{12} & = & s_{0}h\left(k\right)\\ S_{21} & = & s_{0}h^{*}\left(k\right)\\ S_{22} & = & 1 \end{array}$$ (4)

where, *s*_0_ is the dimensionless overlap integral. In this work we have followed the convention (e.g. Saito et al. (1998); McCann (2012)) of fixing the energy origin for the bands by setting the unperturbed energy of the *p*_*z*_-orbital *ϵ*_2*p*_ = 0 (a possible limitation of this convention would be considered elsewhere); we also adopt the parameter values *g*_0_ = 3.033 eV, *s*_0_ = 0.129, and the C-C bond length *a*_*CC*_ = 0.142 nm, quoted therein (with the lattice constant $a=\sqrt{3}a_{CC}$ = 0.246 nm).

Before proceeding further, we note that Eq. 1 is not a proper Schrödinger equation, due to the fact that *S* is not a unit matrix and, hence, *H* ^0^ is not a proper Hamiltonian. We may, however, obtain a proper Hamiltonian by operating Eq. 1 with the inverse of *S*, from the left, and define the present TB Hamiltonian by *H* ≡ *S* ^−1^ *H* ^0^. The associated Schrödinger equation of the system is then given by

$$H\psi_{k}=E\psi_{k}$$ (5)

where the elements of the 2 × 2 Hamiltonian matrix *H*

$$\begin{array}{ccc} H_{11} & = & \left(\epsilon_{2p}+g_{0}s_{0}f^{2}\left(k\right)\right)/\left(1-s_{0}^{2}f^{2}\left(k\right)\right)\\ H_{12} & = & -g_{0}f\left(k\right)e^{i\chi \left(k\right)}/\left(1-s_{0}^{2}f^{2}\left(k\right)\right)\\ H_{21} & = & -g_{0}f\left(k\right)e^{-i\chi \left(k\right)}/\left(1-s_{0}^{2}f^{2}\left(k\right)\right)\\ H_{22} & = & \left(\epsilon_{2p}+g_{0}s_{0}f^{2}\left(k\right)\right)/\left(1-s_{0}^{2}f^{2}\left(k\right)\right) \end{array}$$ (6)

The eigenvalues and the eigenvectors of *H* are easily obtained analytically by diagonalisation:

$$\begin{array}{ccc} E_{1}\left(k\right) & = & \left(\epsilon_{2p}-g_{0}f\left(k\right)\right)/\left(1+s_{0}f\left(k\right)\right)\\ E_{2}\left(k\right) & = & \left(\epsilon_{2p}+g_{0}f\left(k\right)\right)/\left(1-s_{0}f\left(k\right)\right) \end{array}$$ (7)

where, **k** = (*k*_*x*_, *k*_*y*_) is the 2D lattice-momentum in the (*x*, *y*)-plane of the monolayer graphene. The corresponding normalised eigenvectors are,

$$\begin{array}{ccc} \psi_{1}\left(k\right) & = & \frac{1}{\sqrt{2}}\left(1,e^{-i\chi \left(k\right)}\right)\\ \psi_{2}\left(k\right) & = & \frac{1}{\sqrt{2}}\left(-1,e^{-i\chi \left(k\right)}\right) \end{array}$$ (8)

We note that although for graphene the square of *s*_0_ = 0.129 is small compared to 1, it appears in the first order in the denominators in the eigenvalues (7) and so in principle should be retained. These eigenvalues were first obtained by Saito et al. (1998) from the variational equations (without requiring the Hamiltonian (6)). Note also that if the parameter *s*_0_ is neglected (set equal to zero) then, one obtains a pair of symmetric energy bands. This is particularly so near the Dirac points where the interaction with the laser field is dominant. One may note that with *s*_0_ = 0.129 the energy bands are generally asymmetric and the VB and CB have different band widths (cf. e.g. Figure 2). The Hamiltonian matrix, Eq. 6, allows us to obtain also the other physically relevant operators of the system analytically. For example, the current operator **J**_*op*_ or, the transition dipole operator **D**_*op*_, can be obtained in useful analytic forms (see, below).

Introduction of the laser-graphene interaction in the system is readily implemented within the present theory by the *minimal coupling* prescription (see, e.g., Section 1.2, Faisal (1987)) in the reciprocal **k**-space, which, in the dipole approximation, consists in merely changing the Hamiltonian (6) by the simple substitution

$$k\to k_{t}\equiv \left(k-\frac{e}{\hbar c}A\left(t\right)\right)$$ (9)

where, **A**(*t*) is the vector potential associated with the laser electric pulse **F**(*t*), and is given by the definition $A\left(t\right)=-c\int_{0}^{t}F\left(t^{\prime }\right)dt^{\prime }$ . Thus, the time-dependent Schrödinger equation governing the evolution of the interacting laser-graphene system can be written as,

$$i\hbar \frac{d}{dt}\Psi_{k}\left(t\right)=H\left(k_{t}\right)\Psi_{k}\left(t\right)$$ (10)

To solve it, we may first expand the total wavefunction Ψ_**k**_(*t*) in terms of the adiabatic (or,“instantaneous”) basis states *ψ*_1_ (**k**_*t*_) and *ψ*_2_ (**k**_*t*_) of *H* (**k**_*t*_) and write:

$$\Psi_{k}\left(t\right)=c_{1}\left(k,t\right)\psi_{1}\left(k_{t}\right)+c_{2}\left(k,t\right)\psi_{2}\left(k_{t}\right)$$ (11)

We substitute it in Eq. 10 and project on to the two orthonormalized basis states |1⟩ ≡ *ψ*_1_ (**k**_*t*_) and |2⟩ ≡ *ψ*_2_ (**k**_*t*_) from the left to get the pair of coupled equations for the time-dependent occupation amplitudes of the valence and the conduction bands, *c*_1_ (**k**, *t*) and *c*_2_ (**k**, *t*), respectively:

$$\begin{array}{ccc} i\hbar \frac{d}{dt}c_{1}\left(k,t\right)=V_{11}\left(k_{t}\right)c_{1}\left(k,t\right)+V_{12}\left(k_{t}\right)c_{2}\left(k,t\right) & & \\ i\hbar \frac{d}{dt}c_{2}\left(k,t\right)=V_{21}\left(k_{t}\right)c_{1}\left(k,t\right)+V_{22}\left(k_{t}\right)c_{2}\left(k,t\right) & & \end{array}$$ (12)

with,

$$\begin{array}{ccc} V_{11}\left(k_{t}\right) & = & \left(\epsilon_{2p}-g_{0}f\left(k_{t}\right)\right)/\left(1+s_{0}f\left(k_{t}\right)-\frac{1}{2}\frac{d}{dt}\chi \left(k_{t}\right)\right)\\ V_{12}\left(k_{t}\right) & = & \frac{1}{2}\frac{d}{dt}\chi \left(k_{t}\right)\\ V_{21}\left(k_{t}\right) & = & \frac{1}{2}\frac{d}{dt}\chi \left(k_{t}\right)\\ V_{22}\left(k_{t}\right) & = & \left(\epsilon_{2p}+g_{0}f\left(k_{t}\right)\right)/\left(1-s_{0}f\left(k_{t}\right)-\frac{1}{2}\frac{d}{dt}\chi \left(k_{t}\right)\right) \end{array}$$ (13)

Note that a common term, $-\frac{1}{2}\frac{d}{dt}\chi \left(k_{t}\right)$ , that appears in the diagonal matrix elements *V*_11_ and *V*_22_ above, can be transformed away by a unitary transformation of the amplitudes without affecting the probabilities and the expectation values calculated with respect to the total wavefunction of the system (and, so, are dropped in the sequel).

### 2.1 Velocity Operator, Dipole Operator, *Intra*band Current, *Inter*band Current and Total Current

The *x* and *y* components of the velocity operator are obtained in the present theory analytically from the Hamiltonian 6) by differentiation with respect to the components of the lattice-momentum **k** = (*k*_*x*_, *k*_*y*_),

$$\begin{array}{ccc} u_{x}\left(k_{t}\right) & = & \frac{1}{\hbar }\frac{d}{dk_{x}}H\left(k_{t}\right)\\ u_{y}\left(k_{t}\right) & = & \frac{1}{\hbar }\frac{d}{dk_{y}}H\left(k\left(t\right)\right) \end{array}$$ (14)

The quantum mechanical current operator defined as **J**_*op*_ = *e* **u**_*op*_ and the transition dipole operator can be obtained from the velocity operator using the Heisenberg equation of motion:

$$J_{op}=\frac{d}{dt}D_{op}=\frac{i}{\hbar }\left[H,D_{op}\right]$$ (15)

Taking the matrix element of the above equation between a pair of eigenstates, |*ψ*_1_ > and |*ψ*_2_ >, with the respective eigenvalues, *E*_1_ and *E*_2_, of H (6), one finds the useful relation,

$$D_{21}=-i\hbar J_{21}/\left(E_{2}-E_{1}\right)$$ (16)

For the applications, the incident laser field **F**(*t*) may be defined generally in the form $F\left(t\right)={\hat{e}}_{x}F_{x}\left(t\right)+{\hat{e}}_{y}F_{y}\left(t\right)$ , where $\left({\hat{e}}_{x},{\hat{e}}_{y}\right)$ are unit polarisation vectors along the *x* and *y* axes, respectively; the associated vector potential is given by $A\left(t\right)=-c\int_{0}^{t}F\left(t^{\prime }\right)dt^{\prime }$ . The interaction Hamiltonian, in the adiabatic representation, for the transition dipole operator in the so-called “length gauge” is simply,

$$\begin{array}{ccc} H_{int}\left(k_{t},t\right) & = & D\left(k_{t}\right)\cdot F\left(t\right)\\ & = & D_{x}\left(k_{t}\right)F_{x}\left(t\right)+D_{y}\left(k_{t}\right)F_{y}\left(t\right) \end{array}$$ (17)

We point out in passing that the coupled dynamical Eq. 12 derived here using the adiabatic basis representation and the minimal coupling prescription in the so-called “momentum gauge” are, in fact, gauge invariant (cf. Krieger and Iafrate (1986)). This can be ascertained by comparing the respective time-dependent coupled equations in the two gauges (in the present representation) and noticing that the off-diagonal coupling matrix elements *V*_12_ (**k**_*t*_) and *V*_21_ (**k**_*t*_) in (13) are equal to the transition matrix elements of the dipole interaction Hamiltonian above (on performing the simple differential operation, $\frac{1}{2}\frac{d}{dt}=\frac{1}{2}\left(F_{x}\left(t\right)\frac{\partial }{\partial k_{x}}+F_{y}\left(t\right)\frac{\partial }{\partial k_{y}}\right)$ on the phase function *χ*(**k**_*t*_) that appears in Eq. 13).

Finally, we give the expressions for the quantum mechanical *expectation value* of the “current” along the *x* and *y* axes, using the interacting total wavefunction of the system, Ψ(**k**, *t*), and summing over the **k**-states of the first Brillouin zone (BZ) including the 2-fold spin- and valley-degeneracy of graphene, respectively *g*_*s*_ = 2, and *g*_*v*_ = 2:

$$\begin{array}{ccc} \hat{e}\cdot J\left(k,t\right) & = & g_{s}g_{v}\underset{BZ}{\sum }\langle \Psi \left(k,t\right)|e\left(\hat{e}\cdot u_{op}\left(k_{t}\right)\right)|\Psi \left(k,t\right)\rangle \\ & = & g_{s}g_{v}{\left(\frac{L}{2\pi }\right)}^{D}\times \int_{BZ}d^{D}k\left\{|c_{1}\left(k,t\right){|}^{2}\left(\Sigma_{i=x,y}e_{i}\langle 1|u_{i}\left(k_{t}\right)|1\rangle \right)\right.\\ & & +c_{2}\left(k,t\right){|}^{2}\left(\Sigma_{i=x,y}e_{i}\langle 2|u_{i}\left(k_{t}\right)|2\rangle \right)\\ & & \left.+2Re\left[c_{2}^{*}\left(k,t\right)c_{1}\left(k,t\right)\left(\Sigma_{i=x,y}e_{i}\langle 2|u_{i}\left(k_{t}\right)|1\rangle \right)\right]\right\} \end{array}$$ (18)

where $\hat{e}=\left(e_{x},e_{y}\right)$ is the unit polarisation vector. $\sum_{BZ}\equiv {\left(\frac{L}{2\pi }\right)}^{D}\int_{BZ}\left(\cdots \;\right)d^{D}k$ stands for the state-sum in the **k**-space and *L* ^*D*^, for the “volume” in the lattice-space (with D = 2 for the monolayer graphene and D = 1 for the armchair graphene ribbons).

We note that the first two sums on the right hand side above, that depend directly on the occupation probability of the valence band (VB = |1⟩) and the conduction band (CB = |2⟩), is often referred to as the *intra*band current, while the third sum that depends on the VB-CB correlation (or “coherence”) term is referred to as the *inter*band current. It is convenient for most purposes to deal with the corresponding *normalised* currents (normalised per $g_{s}g_{v}{\left(\frac{L}{2\pi }\right)}^{D}$ ) as:

$$j^{total}\left(t\right)=j^{intra}\left(t\right)+j^{inter}\left(t\right)$$ (19)

$$\begin{array}{ccc} j^{intra}\left(t\right) & = & \int_{BZ}d^{D}k\left\{|c_{1}\left(k,t\right){|}^{2}\left(\Sigma_{i=x,y}e_{i}\langle 1|u_{i}\left({\text{k}}_{t}\right)|1\rangle \right)\right.\\ & + & \left.|c_{2}\left(k,t\right){|}^{2}\left(\Sigma_{i=x,y}e_{i}\langle 2|u_{i}\left(k_{t}\right)|2\rangle \right)\right\} \end{array}$$ (20)

$$j^{inter}\left(t\right)=\int_{BZ}d^{D}k\left\{2Re\left[c_{2}^{*}\left(k,t\right)c_{1}\left(k,t\right)\left(\Sigma_{i=x,y}e_{i}\langle 2|u_{i}\left({\text{k}}_{t}\right)|1\rangle \right)\right]\right\}$$ (21)

We note that the normalised currents are in a. u., with $\left[1a.u.\right]=\left(\frac{e}{t_{0}}\right)/a_{0}^{D-1}$ = $0.125\left(\frac{C}{s}\right)$ /nm, for the monolayer (D = 2) and, = $6.624\times 10^{-3}\left(\frac{C}{s}\right)$ for the ribbons (D = 1).

To complete the definitions we also give the matrix elements appearing above, explicitly:

$$\begin{array}{ccc} \langle 1|u_{i}\left(k_{t}\right)|1\rangle & = & -\left(g_{0}+e_{2p}s_{0}\right)\frac{\partial f\left(k_{t}\right)}{\partial k_{i}}{\left(1+s_{0}f\left(k_{t}\right)\right)}^{2}\\ \langle 2|u_{i}\left(k_{t}\right)|1\rangle & = & i\left(g_{0}+e_{2p}s_{0}\right)\partial \chi \left(k\left(t\right)\right)\partial k_{i}f\left(k_{t}\right)/\left(1-s_{0}^{2}f^{2}\left(k_{t}\right)\right)\\ \langle 1|u_{i}\left(k_{t}\right)|2\rangle & = & -i\left(g_{0}+e_{2p}s_{0}\right)\partial \chi \left(k_{t}\right)\partial k_{i}f\left(k_{t}\right)/\left(1-s_{0}^{2}f^{2}\left(k_{t}\right)\right)\\ \langle 2|u_{i}\left(k_{t}\right)|2\rangle & = & \left(g_{0}+e_{2p}s_{0}\right)\frac{\partial f\left(k_{t}\right)}{\partial k_{i}}{\left(1-s_{0}f\left(k_{t}\right)\right)}^{2},i=\left(x,y\right). \end{array}$$ (22)

For the simulations made in this work, we have solved the coupled Eq. 12 numerically to obtain the amplitudes *c*_1_ (**k**, *t*) and *c*_2_ (**k**, *t*). They are used to determine the total wavefunction of the interacting system and to construct the expectation values to investigate first the excitation of the CB population and the *inter*band correlation (or “coherence”). Next, the *intra*band and the *inter*band currents as well as the total current are simulated for the case of graphene monolayer and an armchair ribbon (width, N = 3). Also simulated are the spectra of the radiation emitted from the monolayer and the ribbon currents. Lastly, the effect of the so-called carrier-envelope-phase (or, CEP) on the shape or symmetry of the current produced in a ribbon (width, N = 1) is studied. It is suggested that ultrashort charge-current pulses of desired shape or symmetry might be possible to design by tailoring the *vector potential* of the laser pulse similarly. The results of the simulations carried out are illustrated graphically, and are compared, and discussed in the next section.

## 3 Results and Discussions

Unless stated explicitly otherwise, for the convenience of writing and the numerical simulations, in the rest of the work we have used the Hartree atomic units: *e* = *m* = *ℏ* = 1, *c* = *α* ^−1^ = 137.036. We note also that [1 a. u.] of length = *a*_0_ = 1Bohr = 0.0519 nm [1 a. u.] of time = *t*_0_ = *a*_0_/(*αc*) = 24.19 as, and [1. a. u.] of e = 1.602 × 10^–19^ C.

### 3.1 Band Structure of Graphene and Armchair Graphene Ribbons

**FIGURE 3:**
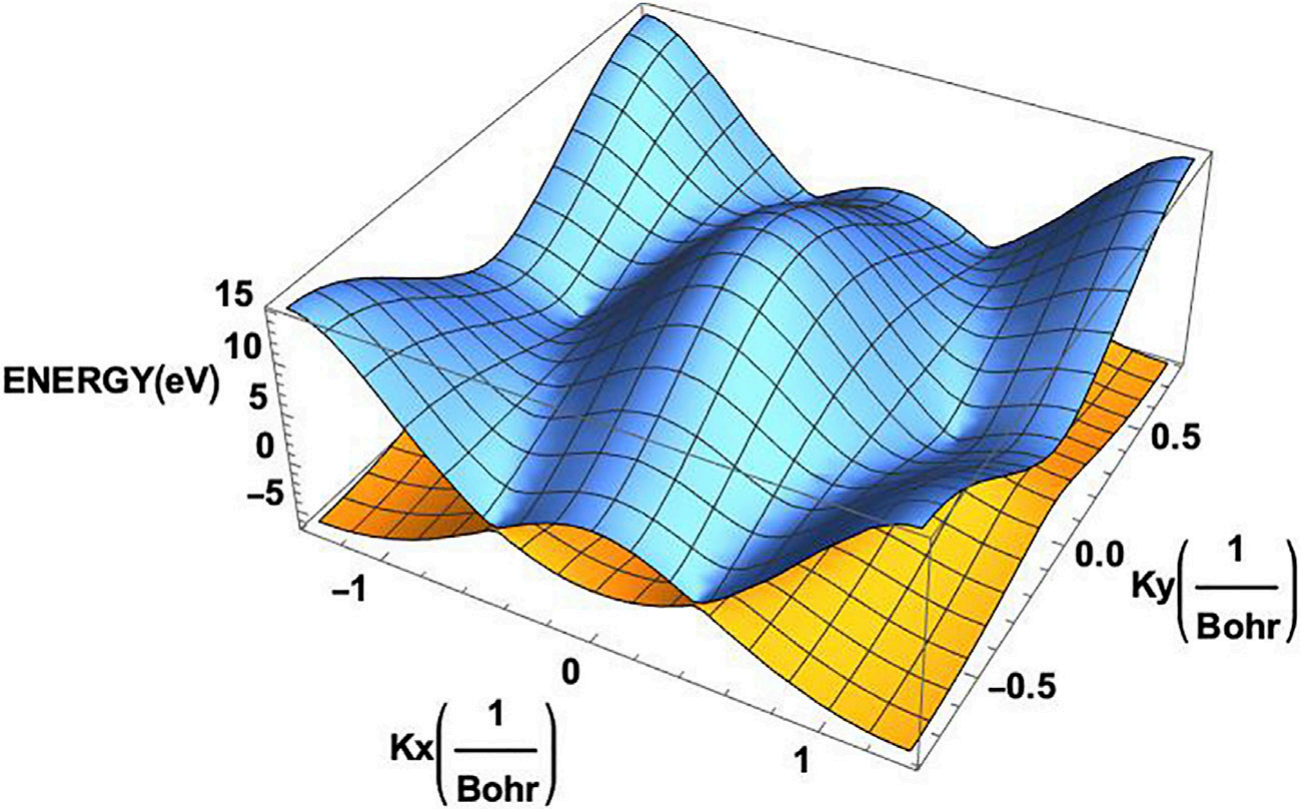
Two dimensional band structure of the monolayer graphene. The valence band (orange) and the conduction band (blue) clearly exhibit the corners of the hexagonal Brillouin zone on them.

In Figure 2, we show a cut through the valence band (VB, blue) and the conduction band (CB, orange) of the monolayer graphene along the *k*_*x*_-axis (for *k*_*y*_ = 0). The energy degeneracy of the two bands are seen to occurr at the Dirac points ( $k_{x}=\pm \frac{4\pi }{3a}=\pm 0.9$ a. u.). Note that in the vicinity of the Dirac points the band dispersions are essentially linear. This is similar to the linear dispersion relation for a hypothetical relativistic free Dirac electron of “zero mass” (hence the nomenclature, “Dirac fermion”). If we neglect the finite overlap integral and set it to *s*_0_ = 0, the present TB Hamiltonian naturally goes over to the usual TB dispersion relations with symmetric band widths of the VB and CB. In Figure 3 we show the full 2D energy surface of graphene (for *s*_0_ = 0.129) in the (*k*_*x*_, *k*_*y*_)-plane where one can also readily recognise the hexagonal structure of the Brillouin zone of the monolayer graphene. In this work, as indicated earlier, we also consider the armchair graphene ribbons cut along the armchair edge (*y*-axis) with a finite number of cell widths, N, along the transverse direction (*x*-axis) (cf. Figure 1). The *confinement* of the ribbon to a finite width along the *x*-axis in fact *quantises* the continuum *k*_*x*_-states of the monolayer into a set of discrete values that depends on the width index N (the number of cells within the width of the “armchair ribbon”) while the ribbon’s length is assumed to extend freely along the armchair axis (*y*-axis).

#### 3.1.1 Rules for Constructing Band Dispersion Relations for a Ribbon of a Given Width

We give below two simple, yet general, rules for obtaining the entire system of energy band dispersion relations of the sub-bands of the armchair graphene ribbons (of any width N):

*Rule 1*: An armchair ribbon of width N has N VB-CB band-pairs, r = 1, 2, … , up to N.

*Rule 2*: The *N* pairs of dispersion relations of the bands can be obtained from the eigenvalues of the monolayer graphene by the simple substitution: *k*_*x*_ → *k*_*x*_ [*N*, *r*] = (2/*a*) (*rπ*/(*N* + 1)), where *a* is the graphene lattice constant).

**FIGURE 4:**
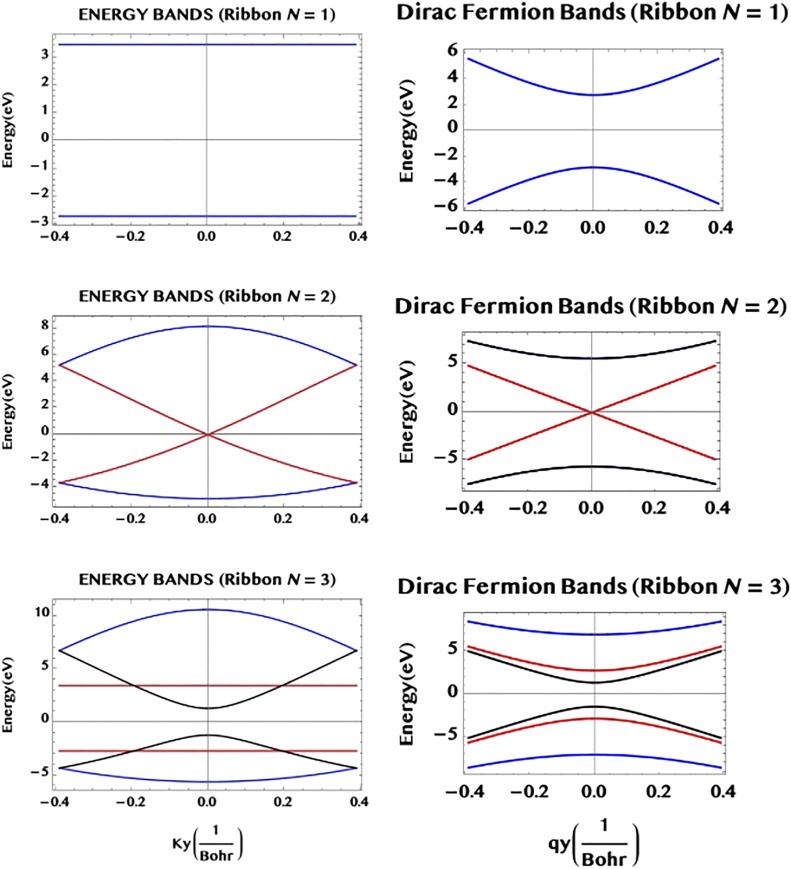
The band structure of armchair graphene ribbons having widths N = 1, 2 and 3, are constructed using the two simple rules for the ribbons given in the text. The full bands from the TB theory (left hand side column), as well as the approximate bands from the Dirac fermion model (right hand side column) are shown. They agree near the middle of the bands (a Dirac point) as they should (but differ away from it). The N = 1 ribbon (“chain”) has a single (r = 1, blue) pair of “flat” bands of constant separation along the armchair axis (horizontal *y*-axis); the N = 2 ribbon has two pairs of VB-CB bands, one pair (r = 2, red) is degenerate at the Dirac point; the N = 3 ribbon has three pairs of VB-CB bands, one pair (r = 2, red) has a constant separation (cf. N = 1), while another pair (r = 3, black) shows a finite band-gap minimum at the centre as in a 1D semiconductor. Note that they follow the “m-rules”, for the occurrence of a degenerate and/or a “flat” pair of sub-bands, as given in the text.

**FIGURE 5:**
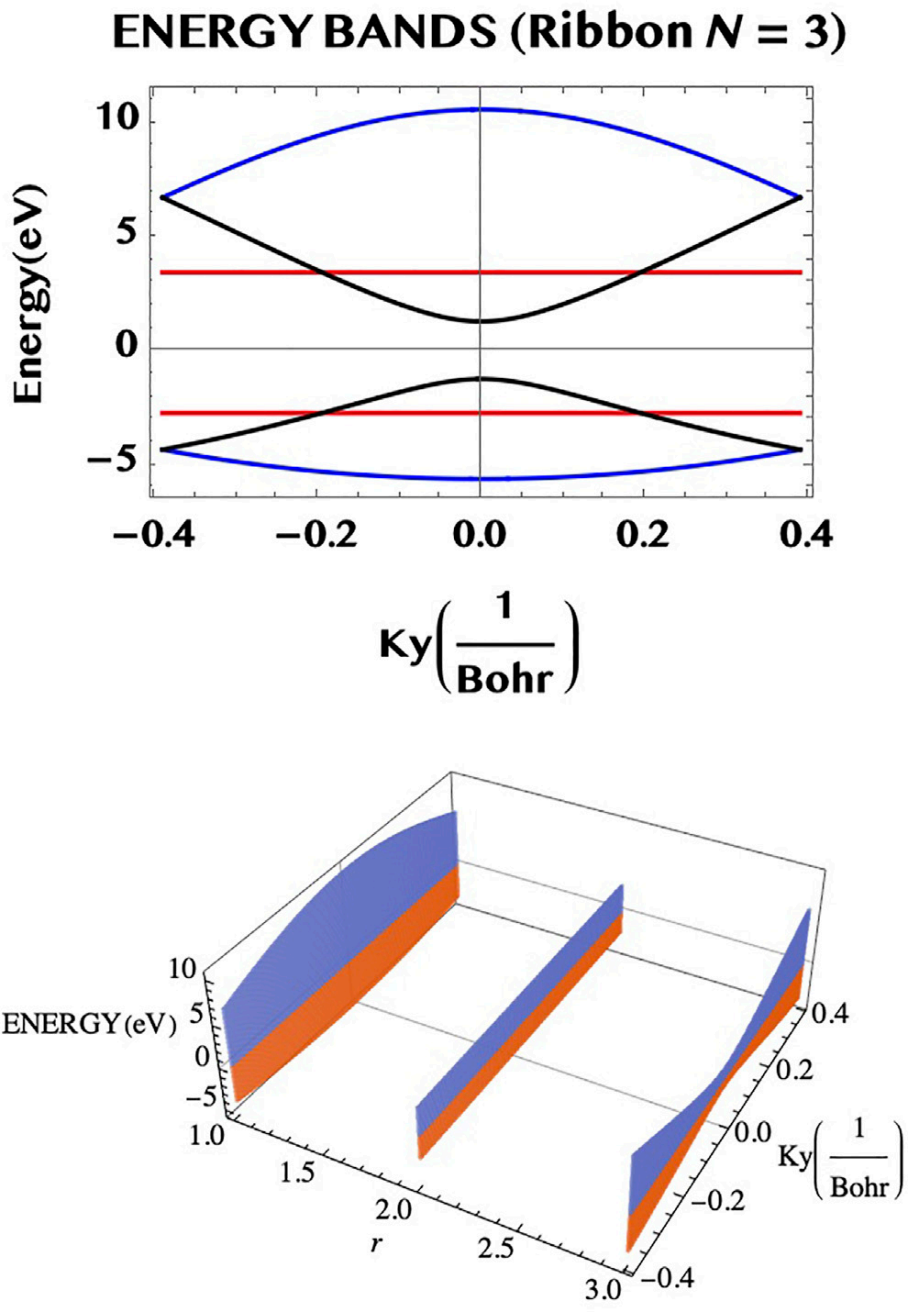
The dispersion relation of the N = 3 armchair ribbon (upper part) may be seen from a different perspective (lower part) as consisting of three “channels” of VB–CB pairs (along the armchair axis), separated by unit widths in the transverse direction.

They apply to the full TB dispersion relations as well as to the Dirac fermion model (applicable near the Dirac points **K**). Thus, for the Dirac fermion model of armchair ribbons we get the following simple dispersion relations of the *r*th pair of bands of a ribbon of width N:

$$\begin{array}{ccc} E_{\pm }\left(N,r,q_{y}\right)=\pm v_{F}\sqrt{q_{x}{\left[N,r\right]}^{2}+q_{y}^{2}}, & & \\ q_{x}\left[N,r\right]\equiv \left(k_{x}\left[N,r\right]-\frac{4\pi }{3a}\right),q_{y}\equiv k_{y} & & \end{array}$$ (23)

where, the Dirac fermion velocity *v*_*F*_ ≈ *c*/300. It is also worth observing (cf. Karakachian et al. (2020)) that 1) for each N = 2m + 2 (for, m = 0, or, integer), there is a degenerate pair of VB-CB bands (with a zero band gap) and 2) for each N = 2m + 1 (m = 0, or, integer), there is a “flat” pair of VB-CB bands, for which the band-gap remains *constant* throughout the *k*_*y*_-space. We may point out that such a ribbon with a flat pair of bands provides a large number of identical “two level” systems (rather analogous to the atomic “two level” systems but) in a robust and compact form of an armchair ribbon. This might be of interest for potential applications in digital/optical systems. In Figure 4 we illustrate the band structure of the armchair ribbons of widths N = 1, 2, and 3, as constructed from the above rules applied to the TB eigenvalues for graphene monolayer (left hand side column) and compare them to the Dirac fermion model, Eq. 23, (right hand side column). It can be seen that both the models agree near the Dirac point, *q*_*y*_ = 0, as they should (but differ elsewhere). The system of VB-CB band pairs are colour coded as follows: (N = 1, r = 1) → blue; (N = 2, r = 1, 2) → (blue, red) and, (N = 3, r = 1, 2, 3) → (blue, red, black). For example, for N = 3, there are three VB-CB pairs of bands none of which is degenerate. However, one pair (red) shows a band-gap minimum, as in a 1D semiconductor. The “m-rules” 1) and 2) given above can be easily verified (for m = 0 or, 1) for the three ribbons of widths N = 1, 2, and 3, shown in the figure. (Not surprisingly, however, the Dirac fermion model, that applies near the Dirac point (*q*_*y*_ = near 0), does not maintain the parallel separation away from the band centre, *q*_*y*_ = 0, unlike the “flat” bands of the full TB model, that do.) We may add that the band structure of the armchair ribbons of width N may be also viewed as possessing N “conduction channels”. Figure 5 illustrates this for N = 3. The bands shown in the upper part of the figure viewed from an alternative perspective (lower part of the figure) helps to visualise the three “channels” along the armchair axis (for r = 1, 2, and three along the width axis).

### 3.2 Interaction With an Intense Ultrashort Laser Pulse

**FIGURE 6:**
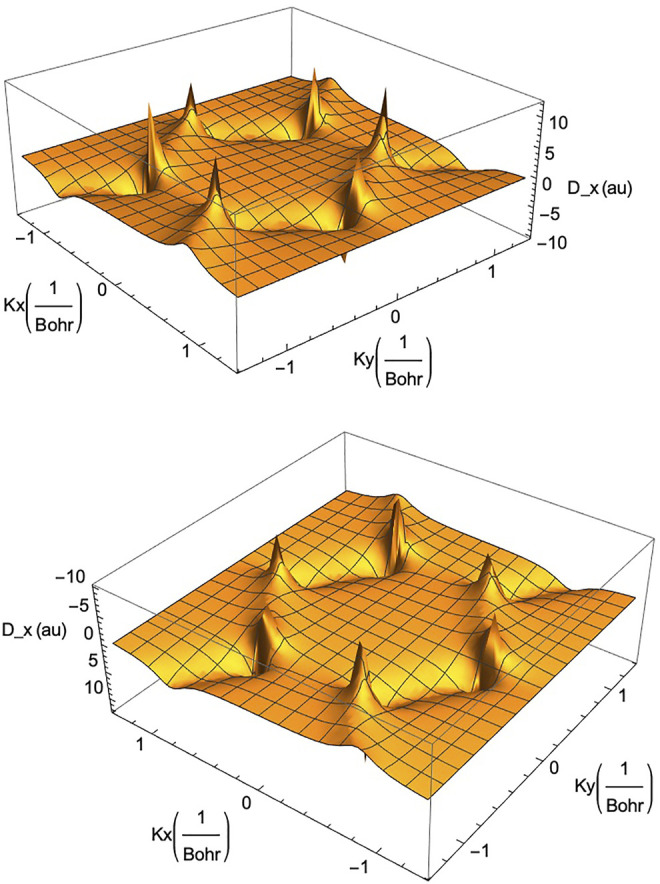
The transition dipole moment operator for monolayer graphene along the *x*-axis, *D*_*x*_ (*k*_*x*_, *k*_*y*_), in the reciprocal space (*k*_*x*_, *k*_*y*_)-plane. Note the high positive values (upper part) and high negative values (lower part) at the corners of the Brillouin zone. A similar behaviour holds for the component of the dipole operator along the *y*-axis, *D*_*y*_ (*k*_*x*_, *k*_*y*_) (not shown here).

As we have seen above, the availability of the TB Hamiltonian in the analytic form (6) allows one to obtain the physically interesting current operator **J**_*op*_ = *e* **u**_*op*_ (below, Eq. 14) and the dipole operator **D**_*op*_ Eq. 16) for the graphene system. They control the response of graphene and graphene ribbons to laser fields. In Figure 6 we show an example of the x-component of the transition dipole operator of graphene as a function of the lattice momentum **k** = (*k*_*x*_, *k*_*y*_). It is clear from the figure that the strength of the transition moment is particularly strong near the corners of the hexagonal BZ (or the Dirac points) in both positive (upper part in the figure) and negative (lower part of the figure) signs of the strength.

For the simulations in this work we have generally restricted ourselves to an intensity of 1 TW/cm^2^ (or, a peak field strength *F*_0_ = 2.72 V/nm that is reasonably high but is below the damage threshold for a monolayer graphene Currie et al. (2011). More specifically, we have chosen a “sin^2^-envelope” for the electric field, **F**(*t*), polarised linearly and parallel to the “armchair edge” of graphene (the *y*-axis):

$$F\left(t\right)={\hat{e}}_{y}F_{0}\sin^{2}\left(\pi \frac{t}{t_{p}}\right)\cos\left(\omega t+\phi_{0}\right)$$ (24)

where, *F*_0_ is the peak electric field strength, *t*_*p*_ is the pulse duration, *ω* is the circular frequency and *ϕ*_0_ is the carrier-envelope-phase (or CEP). The corresponding vector potential is given by,

$$A\left(t\right)=-c\int_{0}^{t}F\left(t^{\prime }\right)dt^{\prime }$$ (25)

**FIGURE 7:**
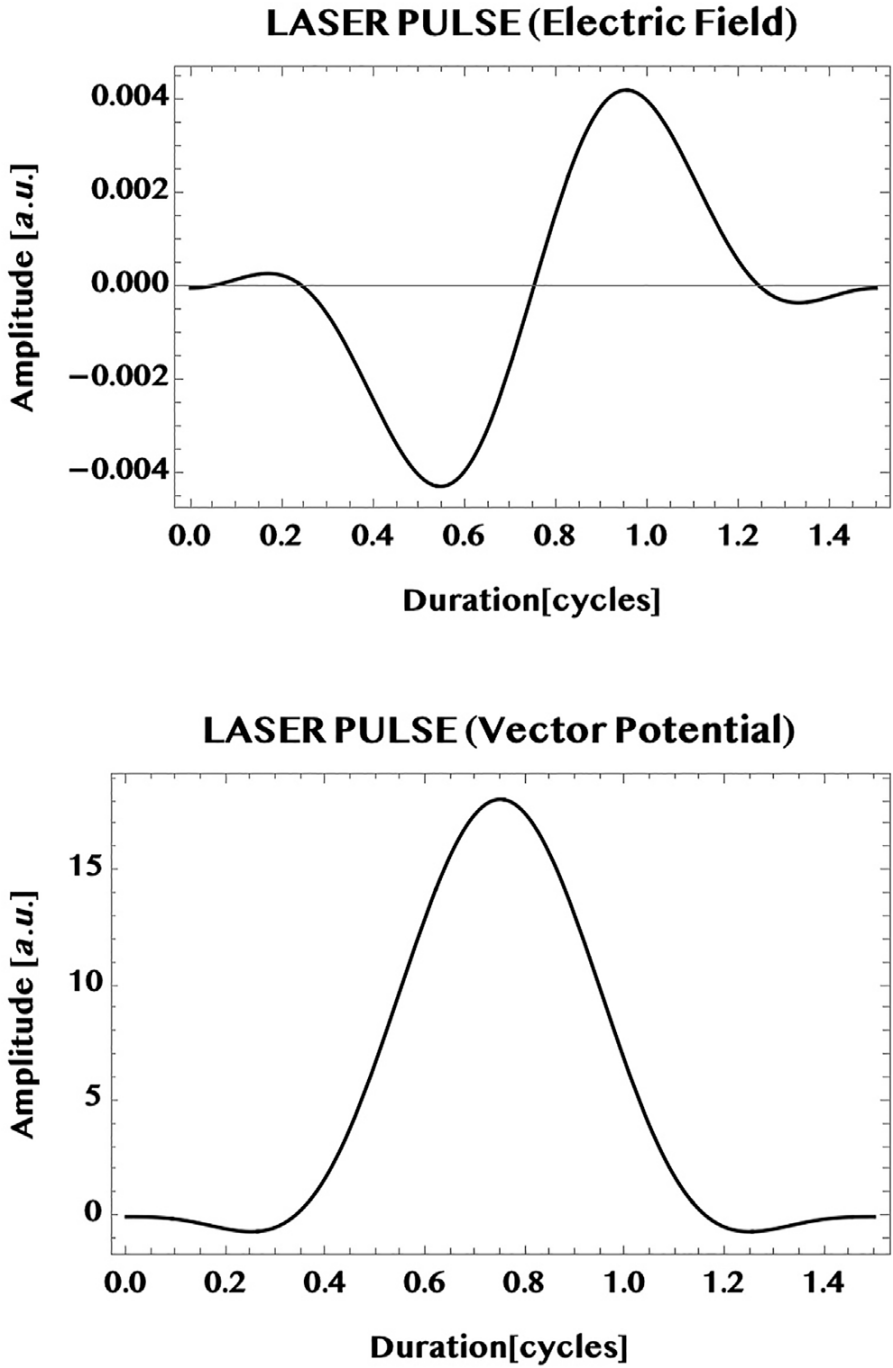
Illustration of a typical ultrashort near-infrared laser pulse used for the simulations in this work (wavelength = 800 nm, peak intensity = 1 TW/cm^2^, CEP = 0, pulse duration = 1.5 cycles). Shown in the upper part is the “sin^2^-envelope” electric field and in the lower part, the corresponding vector potential. Vertical axis: pulse amplitude in a. u. (field strength [1 a. u.] = 514.22 V/nm), horizontal axis: pulse duration in cycles (1 cycle = T = 2*π*/*ω*): The pulse chosen is linearly polarised parallel to the armchair edge (*y*-axis): $F\left(t\right)={\hat{e}}_{y}F_{y}\left(t\right)={\hat{e}}_{y}F_{0}\sin^{2}\left(\pi \frac{t}{t_{p}}\right)\cos\left(\omega t+CEP\right)$ , where $\hat{e}$ = unit polarisation vector, *F*_0_ = peak field strength, *t*_*p*_ = pulse duration, *ω* = circular frequency, CEP = carrier-envelope-phase. The corresponding vector potential is $A_{y}\left(t\right)=-c\int_{0}^{t}F_{y}\left(t^{\prime }\right)dt^{\prime }$ , c = speed of light in vacuum. For the simulations in this work generally CEP = 0 is used (except for Figure 12, where the CEP-dependence of the current in an armchair ribbon is investigated).

The pulse is illustrated graphically in Figure 7 (upper panel: electric field, lower panel: vector potential; wavelength 800 nm, intensity one TW/cm^2^, *t*_*p*_ = 1.5 cycles or, 4 fs).

Note that the duration of the simulation times are extended mostly up to twice the pulse duration (up to three cycles) with no field present in the last 1.5 cycles. This allows one to observe the residual response of the system *after* the pulse has ended. Simulations are made for the following quantities: 1) CB population, 2) “VB-CB correlation”, and 3) “band currents”, and the results are presented graphically below. They are *normalised*, as discussed above per unit “volume” (precisely, per $g_{s}g_{v}{\left(\frac{L}{2\pi }\right)}^{D}$ , where, *g*_*s*_ = 2, *g*_*v*_ = 2 are the spin and valley degeneracy factors of graphene; D = 2 for the monolayer and, D = 1 for the ribbons). As indicated above, the units of the quantities represented are in Hartree atomic units (a.u.) (with [1a.u.] of length = 0.0529 *nm* [1 a. u.] of time = 24.19 *as*, and [1 a. u.] of charge e = 1.602 × 10^–19^ C). The *normalised* population transfer to the CB and the VB-CB correlation are computed assuming an initially fully occupied VB and an empty CB. Similar initial conditions are assumed for the simulations of the normalised band currents. They are computed from the following normalised formulas (obtained from the general formulas given above).

1) Normalised CB population:

$$Population\left[t\right]=\int_{BZ}|c_{2}\left(k,t\right){|}^{2}d^{D}k\left[a.u.\right],$$ (26)

2) Normalised VB-CB correlation:

$$Correlation\left[t\right]=\int_{BZ}2Re\left[c_{2}{\left(k,t\right)}^{*}c_{1}\left(k,t\right)\right]d^{D}k\left[a.u.\right],$$ (27)

The units for 1) and 2): $\left[1a.u.\right]=\frac{1}{a_{0}^{D}}$ or, 357.3 $\frac{1}{nm^{2}}$ for monolayer graphene (D = 2), and =18.904 $\frac{1}{nm}$ for the ribbons (D = 1).

3) The normalised Currents: They are simulated from the y-component of the formulas, Eqs 19, 20 and 21. The units of the normalised currents are in a. u. with [1*a*.*u*.] = $\frac{e}{t_{0}a_{0}^{\left(D-1\right)}}$ ; D = 2, monolayer, D = 1, ribbons; the conversion factors to S.I. are the same as given below Eq. 21 above.

**FIGURE 8:**
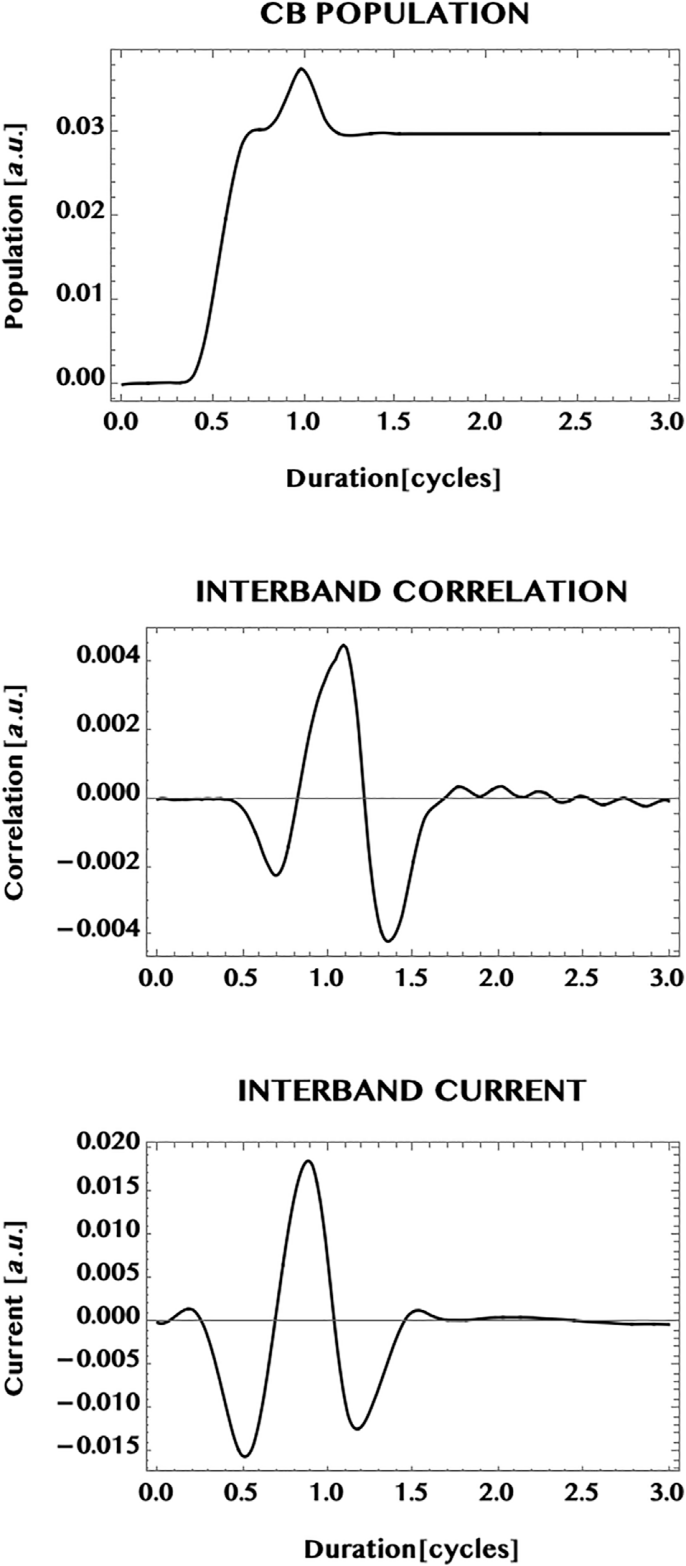
Response of a graphene monolayer to an intense ultrashort laser pulse. Pulse parameters are: wavelength = 800 nm, peak intensity = 1 TW/cm^2^, pulse duration = 1.5 cycles, CEP = 0; simulation period = 3 cycles. Top panel: normalised population excited in the conduction band (CB); middle panel: normalised *inter*band “coherence” (or correlation). Shown also is the normalised *inter*band current (bottom panel). Note the near steady population in the CB and an oscillatory *inter*band coherence after the pulse is over. The *inter*band current (being a 2D integrated sum of the *inter*band coherence *wighted* by the k-dependent current operator) shows only a mild modulation for the 2D monolayer (in comparison with a 1D ribbon that is confined along the width dimension (cf. also Figures 9, 10 below).

**FIGURE 9:**
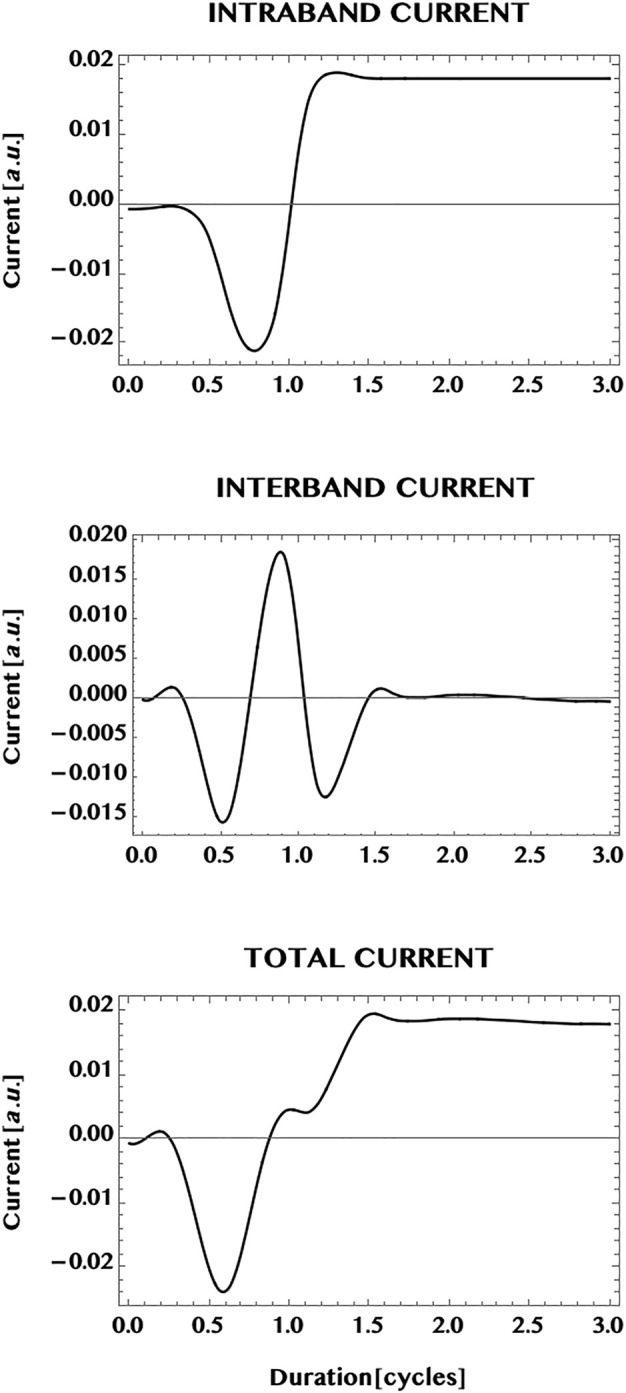
Normalised currents induced in a monolayer graphene by an ultrashort laser pulse (with the same pulse parameters as in Figure 7). The upper panel: *intra*band current; the middle panel: *inter*band current. The total current is shown in the bottom panel. It is dominated by the *intra*band current that shows a near steady state by the end of the pulse, at 1.5 cycles, and after the pulse is over. The mild modulation seen to be present could be an effect of the reduced “coherence” (or correlation) of the *inter*band current by the 2D dispersion effect of the integrated sum of **k**-dependent *inter*band coherence weighted by the 2D current operator. This should be contrasted with the possible effects of “quantum confinement” along the width dimension and the band-gap minimum, in the case of the armchair ribbon (cf. Figure 10).

In Figure 8 we show the population transferred to the CB of graphene (upper panel), the *inter*band correlation—or the VB-CB “coherence”—(middle panel), as well as the *inter*band current (bottom panel). The simulation duration is extended to twice the pulse duration (1.5 cycles) to 3.0 cycles. This allows one to observe the behaviour of the response after the pulse is over. The CB population is seen to increase considerably with the passage of the pulse and attains essentially a steady state superimposed by a mild modulation that persists after the end of the pulse. This is to be contrasted with the oscillatory behaviour of the *inter*band correlation (or the VB-CB “coherence”) that shows a sub-cycle oscillation at the end of the pulse and beyond. We point out that sub-cycle oscillations and ballistic currents have been studied and observed experimentally earlier by Hommelhoff and collaborators (Higuchi et al. (2017); Heide et al. (2020)). Note that the *inter*band current—that corresponds to the *inter*band correlation *weighted* by the current operator—is seen here to show only a mild modulation about zero-current. The difference between the strong oscillation in the *inter*band coherence and the mild modulation of the *inter*band current might be an effect of the weighted dispersion in the two dimensional **k**-space for the *inter*band current compared to the un-weighted *inter*band correlation. In Figure 9 we show the full current (bottom panel) in graphene as well as the individual contributions of the *intra*band current (upper panel) and the *inter*band current (middle panel). Conceptually, the *intra*band current is associated directly with the sum of the electron-current in the CB and the “hole”-current in the VB. The *inter*band current (middle panel) is associated with the *inter*band correlation or the VB-CB “coherence” (discussed above with respect to Figure 8). It can be seen here that the total current (bottom panel) is dominated by the *intra*band current (top panel) and reaches essentially a steady state with a mild modulation. Note that the mild modulation of the *inter*band current causes the mild modulation of the total current beyond the duration of the 1.5 cycle laser pulse.

**FIGURE 10:**
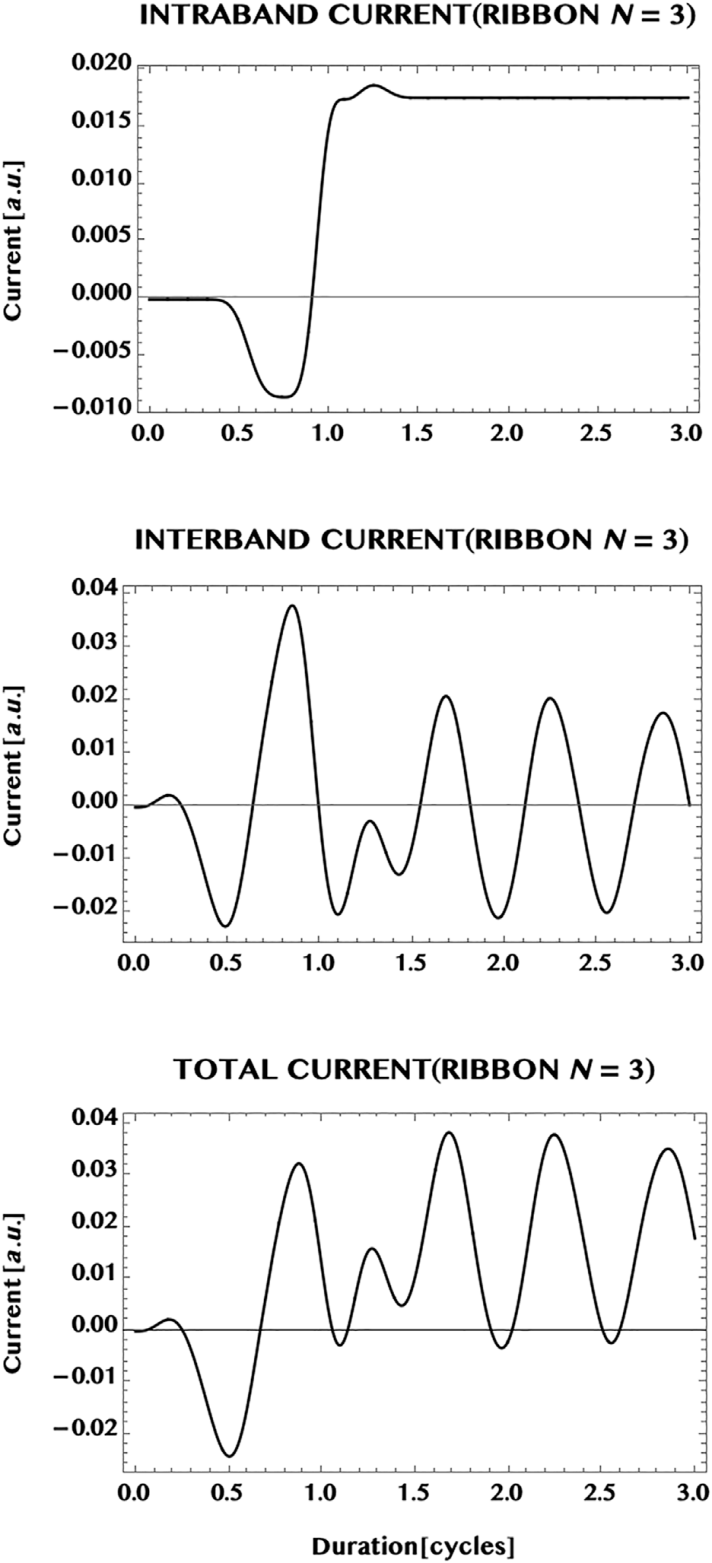
Normalised current generated in an armchair ribbon (N = 3) by an ultrashort laser pulse (wavelength = 800 nm, intensity = 1 TW/cm^2^, pulse duration = 1.5 cycles, CEP = 0; simulation period = 3 cycles). Top panel: *intra*band current; middle panel, *inter*band current; bottom panel: *total* current. Note that the total current is dominated by the *inter*band (“coherent”) current that shows an oscillatory behaviour during and after the pulse. This might be understood as an effect of the *confinement* of the ribbon along the width dimension, resulting in a reduced band dispersion in the 1D ribbon compared to the fully open 2D monolayer.

Next, we compare the currents in the monolayer graphene with the current in an armchair graphene ribbon. In Figure 10 we show the current generated in a ribbon of width N = 3, having three pairs of VB-CB bands or, three “conduction channels” (cf. Figure 5). The laser pulse chosen is the same as in the case of the monolayer i.e. a Ti-Sapphire laser pulse at 800 nm, with a peak intensity of one TW/cm^2^, and a pulse duration of 1.5 cycles. In comparison with the monolayer case (Figure 9), the transition to the ribbon shows a remarkable change of the dominance from the *intra*band current in the 2D monolayer to a dominance of the *inter*band current in the 1D ribbon. The ribbon appears to reduce the possible influence of band dispersion in 1D compared to the open 2D monolayer. This difference might be a result of the confinement effect as well as of the difference in the band structure of the ribbon that has a finite band-gap minimum. However, the same transition from a steady state (with a mild modulation) in the monolayer current to the strong oscillation of the current in the ribbon does not hold universally for the ribbons (as can be seen (cf. Figure 12) from the steady zero-current, albeit at a much reduced laser intensity = 1 GW/cm^2^, in a ribbon of unit width having a pair of “flat” bands with a wide separation, and needs further investigations for a greater clarity.

The induced currents in graphene and the graphene ribbon imply emissions of radiation. We, therefore, have also briefly considered the emission spectra radiated by them.

**FIGURE 11:**
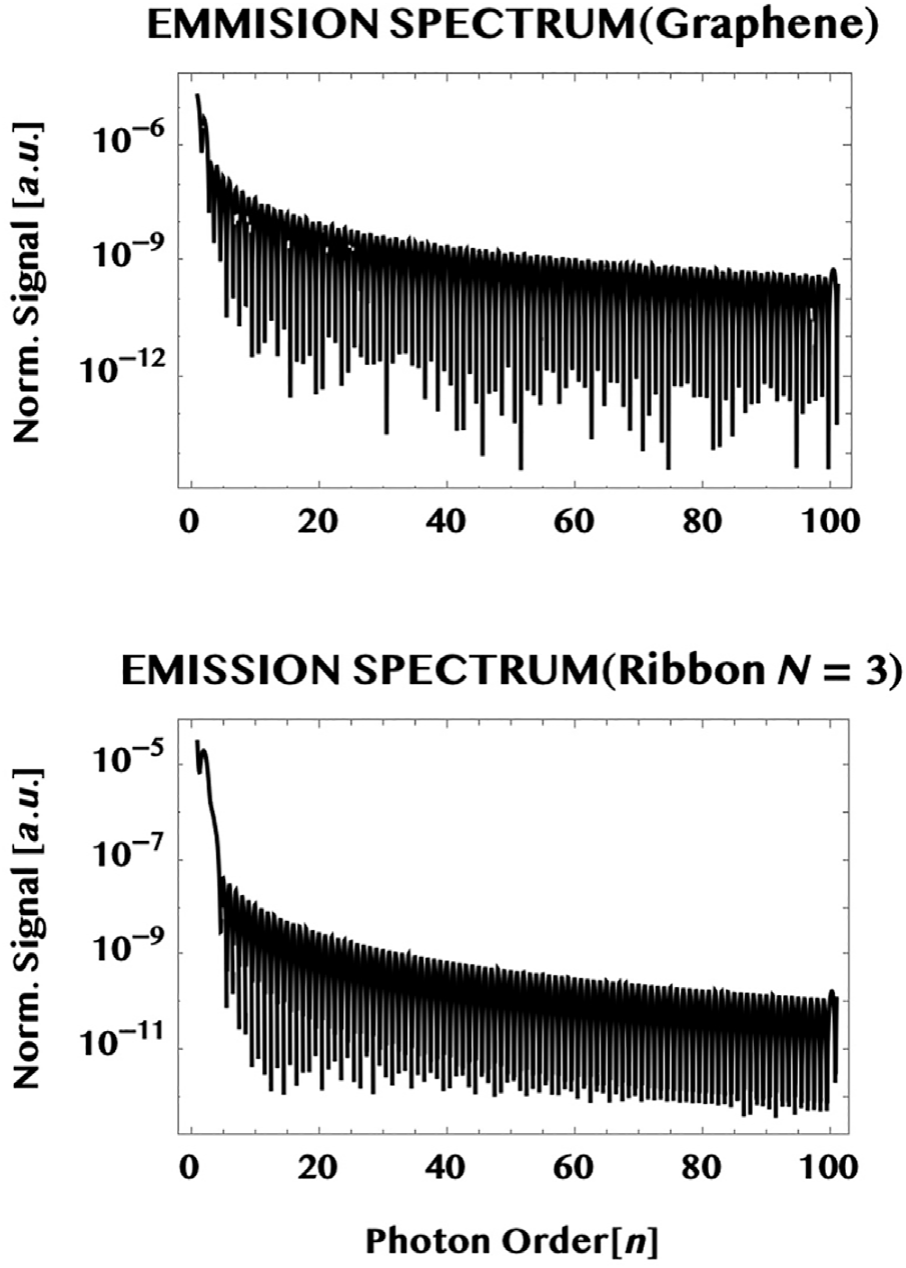
Emission spectra radiated by the ultrashort charge-currents generated in the monolayer graphene (upper plot) and in a graphene ribbon (width, N = 3) (lower plot) by a 1.5 cycles, 800 nm, one TW/cm^2^ laser pulse polarised linearly parallel to the armchair edge (*y*-axis). Notice that both the spectra exhibit similar characteristics i.e. a rapid fall in intensity within a few photon orders followed by a low but broad plateau that extends over many orders (over one hundred).

The normalised emission spectra computed here are defined by the frequency transform of the normalised currents as follows:

$$Signal\left(n\right)=|\frac{1}{t_{p}}\int_{0}^{t_{p}}j_{y}^{total}\left(t\right)e^{-in\omega t}dt{|}^{2}\left[a.u.\right],$$ (28)

where, *t*_*p*_ is the pulse duration. (We may add that the unit of the normalised emission signal in the figure is in [a.u.] and, in S.I. it is simply the square of the units of the normalised currents given earlier.) We show in Figure 11 the normalised spectra of radiation emitted from a graphene monolayer as well as by an armchair ribbon (of width N = 3) interacting with a 1 TW/cm^2^, 800 nm, 1.5 cycle laser pulse (as in Figure 7). The laser is assumed to be incident transversely to the monolayer and polarised linearly along the armchair edge (*y*-axis). It can be seen that both the spectra have similar qualitative characteristics—namely, a high signal for the low photon orders with a rapid fall in intensity followed by a low and *broad* plateau that extends to large orders (over a hundred) of the incident photon energy. They show a relative insensitivity to the detailed structure of the target chosen. This is reminiscent of the relative insensitivity also of the gross structure of the so-called “HHG” spectra of atoms/molecules. From the quantum point of view, this insensitivity of the gross structure of the spectra seen here might be due to the dominance of the virtual continuum-continuum transitions between the two band-continua, like that in the ionisation continuum of atoms/molecules that lie behind the fundamental HHG emission process [cf. e.g., review (Section 4.7), Becker and Faisal (2005)].

**FIGURE 12:**
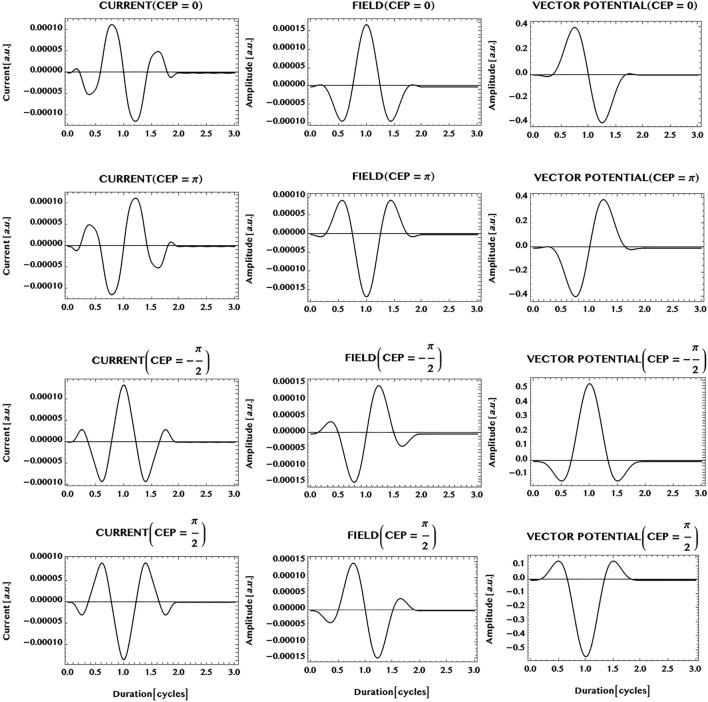
Carrier-envelope-phase (or, CEP) dependence of an ultrashort charge-current generated in an armchair ribbon of unit width (N = 1) by an ultrashort laser pulse (wavelength = 800 nm, peak intensity = 1 GW/cm^2^, pulse duration = 1.5 cycles, simulation period = 3 cycles). Shown are: charge-current (LHS column); electric field (middle column); the vector potential (RHS column). Note that CEP = 0 (row 1), CEP = *π* (row 2), CEP = -*π*/2 (row 3), and CEP = *π*/2 (row 4). It can be seen that the *phase* of the charge-current pulse is correlated with the CEP of the laser pulse. For example, the charge-current pulse is fully *reversed* by changing the CEP = 0 (row 1) to CEP = *π* (row 2). More generally, the shape or symmetry of the main portion of the current is seen to be similar to the shape or symmetry of the *vector potential* of the laser pulse. This suggests the possibility of shaping pulses of charge-carriers to a desired form by tailoring the *vector potential* of the laser pulse similarly.

Finally, we consider the possibility of shaping the ultrashort current of charge carriers in an armchair graphene *ribbon* by tailoring the laser pulse. It has been found recently by Hommelhoff and collaborators that the electron dynamics in monolayer graphene can be controlled by choosing the field strength and the phase of the laser field. Thus, they have observed sub-cycle Stuekelberg oscillations and field dependent change of directions of the ballistic current in monolayer graphene (Higuchi et al. (2017); Heide et al. (2020)). Here we have made a brief study of the influence of the carrier-envelope-phase (CEP) of the laser pulse on the shape of the charge-current pulse generated in an *armchair ribbon*. The results of simulations of the currents (LHS column) are shown in Figure 12, along with the electric field (middle column) and the vector potential (RHS column), for four different carrier-envelope-phases of the laser pulse: CEP = 0, *π*, -*π*/2, and *π*/2. Comparing the top two rows of the figure, with CEP = 0 and CEP = *π*, it can be seen that the symmetry of the ultrashort current pulse is fully *reversed* on changing the CEP of the incident laser pulse from 0 to *π*. More generally, the results show that the *main* part of the currents for different CEPs in the ribbon follow the shape of the *vector potential* of the incident laser pulse. This suggests the possibility of shaping an ultrashort pulse of charge-carriers in the ribbon to a desired form by tailoring the *vector potential* of the incident laser pulse to mimic the form.

## 4 Summary

A quantum mechanical investigation of the interaction of an ultra-short intense laser pulse with a two dimensional monolayer graphene *and* with armchair graphene ribbons of different widths is made. A TB model is used for the graphene bands and two simple rules for generating the system of sub-bands of the armchair graphene ribbons of any width, N, are given and the band structures are discussed with illustrations.

Simulations are carried out to investigate, first, the laser pulse excitation of the CB population, the VB-CB *inter*band “correlation” (or “coherence”) and the currents generated in the monolayer graphene and in an armchair graphene ribbon (width, N = 3). It is found that the population transfer from an initially fully occupied valence band (VB) to an empty conduction band (CB), is seen to increase during the pulse and ends with a finite steady population, that persists after the pulse is over. In contrast the excitation of the *inter*band correlation or, the VB-CB “coherence”, shows an oscillatory behaviour both during and after the passage of the pulse.

A comparison of the currents generated in the monolayer graphene with the current in an armchair ribbon (of width N = 3) shows that the former is dominated by the *intra*band component, that leads to a near steady rest-current with a mild modulation. The transition to the ribbon exhibits a remarkable change from the dominance of the *intra*band current in the monolayer to the dominance of the oscillatory *inter*band current in the ribbon. The ribbon appears to reduce the possible effect of band dispersion in 1D, compared to the open 2D monolayer. The difference seen could be a combined result of the quantum “confinement” effect to 1D as well as due to the difference in the band structure of the ribbon having a band-gap minimum. However, the transition to the oscillatory ribbon current does not hold universally for the ribbons of different widths and, thus, remains open to further investigations in the future for greater clarity.

A brief comparison of the radiation emitted by the currents in the monolayer graphene and the ribbon (N = 3) is also made. They show a gross similarity and a relative insensitivity to the detailed structure of the targets used. The emission spectra are found to be virtually continuous in frequency and fall off rapidly in intensity with the initial photon orders and reach a low but *broad* plateau that extends over many (over a hundred) orders of the incident photon energy. The form of the spectra are also rather insensitive to the target chosen. This is reminiscent of the gross structure and the relative insensitivity to the target chosen also for the well-known “HHG” spectra of atoms or molecules. From the quantum point of view, this insensitivity might be due to the dominance of the virtual continuum-continuum transitions between the two bands, not unlike the transitions in the ionisation continuum of atoms and molecules, that govern the fundamental HHG emission process.

Finally, the possibility of controlling the shape of the ultrashort current of the charge carriers in an armchair ribbon of unit width (N = 1) by the incident laser field is briefly studied. Simulations with different carrier-envelope-phase (CEP) of the incident laser pulse show, for example, that the symmetry of the current in the ribbon can be fully reversed by changing the CEP of the laser pulse from 0 to *π*. More generally, the result of the simulations made shows that the *main* part of the pulse of the charge-carriers in the ribbon follows the shape of the *vector potential* of the incident laser pulse. This suggests the possibility of *shaping* the ultrashort pulse of charge carriers in the ribbon to a desired form by tailoring the *vector potential* of the laser pulse to mimic the form.

## Data Availability

The original contributions presented in the study are included in the article, further inquiries can be directed to the corresponding author.
